# Ultra structural characteristics of methicillin resistant *Staphylococcus aureus* cell wall after affecting with lytic bacteriophages using atomic force microscopy

**DOI:** 10.22038/ijbms.2019.31226.7521

**Published:** 2019-03

**Authors:** Golnar Rahimzadeh, Pooria Gill, Mohammad Sadegh Rezai

**Affiliations:** 1 Pediatric Infectious Diseases Research Center, Mazandaran University of Medical Sciences, Sari, Iran; 2 Student Research Committee, Mazandaran University of Medical Sciences, Sari, Iran; 3 Nanomedicine Group, Immunogenetics Research Center, Mazandaran University of Medical Sciences, Sari, Iran

**Keywords:** AFM, Bacteriophages, Lytic activity, MRSA, 3D topography

## Abstract

**Objective(s):**

During the last years with increasing resistant bacteria to the most antibiotics bacteriophages are suggested as appropriate treatment option. To investigate lytic activity of bacteriophages there are indirect microbial procedures and direct methods. The present study to complement microbial procedures and investigate ultra-structural characteristics of infection bacterium-phage use atomic force microscopy technique.

**Materials and Methods:**

The* Siphoviridae* bacteriophages were isolated from sewage at the Tertiary Pediatric Hospital. Bacteriophages (10×10^8^ PFU/ml) were diluted and were mixed with 100 μl of methicillin resistant *Staphylococcus aureus* (MRSA) ATCC 33591 (1.5×10^8^ CFU/ml). The tubes were incubated for 20 min at 37 ^°^C, at intervals 10 min, 10 μl samples were removed and directly were investigated MRSA ATCC morphology, roughness parameter, 3D topography, cell height, and fast Fourier transform (FFT) by atomic force microscopy (AFM) technique. Concurrently turbidity assay were performed.

**Results:**

Concentration of MRSA ATCC No. 33591 strain after 10 min in phage-treated MRSA S3 (1.5×10^6^ CFU/ml), S4 (1.5×10^5^ CFU/ml), S5 (1.5×10^4^ CFU/ml), S6 (1.5×10^3^ CFU/ml) decreased 2-log, 3-log, 4-log, and 5-log respectively. The results AFM micrographs shown the most changes in bacterial morphology and 3D topography, destruction of cell wall, decrease of cell height, and loss of their shape after 10 min at phage-treated MRSA S3 (1.5×10^6^ CFU/ml), S4 (1.5×10^5^ CFU/ml), S5 (1.5×10^4^ CFU/ml), S6 (1.5×10^3^ CFU/ml) respectively .

**Conclusion:**

In this study MRSA ATCC ultra-structural changes in phage-treated MRSA ATCC groups directly were detected using AFM technique.

## Introduction

Methicillin-resistant *Staphylococcus aureus* (MRSA) is Gram-positive that causes variety of human infections from abscesses, endocarditis, pneumonia to fatal sepsis. MRSA strains frequently exhibit resistance to variety of common antibiotics (1-4).

In the last few years multidrug resistance bacteria (MDR) have increased and have return infectious diseases. Bacteriophages are suggested as an alternative therapeutic strategies. Characteristics of bacteriophage include: specific lytic activity against MDR strains, non-destruction eukaryotic cells and normal flora, and have not reported resistance to them (5-9).

To investigate interaction bacterium-phages use conventional microbial methods such as double-layer plaque assay (DLA), spot test, turbidity concentration which lytic activity of phages in above methods detect with formation of plaque, zone of inhibition, and reduction of bacterial concentration at long time. The results of conventional microbial methods may be affected by environmental factors such as pH, temperature, etc. (10-14). 

To direct investigate interaction bacterium-phages perform transmission electron microscopy (TEM), scanning electron microscopy (SEM), and atomic force microscopy technique.

Electron microscopies (EMs) detect morphology and size with resolution >10 nm, and do not detect biological specimen roughness and 3D topographic in biological specimens. 

Atomic force microscopy (AFM) is a type of scanning probe microscopy (SPM) based on measurement forces between the probe and sample surface which detects morphology and size with resolution <10 nm, roughness and 3D topographic data , force spectroscopy measurements and mechanical attributes at the molecular level (15, 16). AFM micrographs show three-dimensional shape (topography) based on position and height probe in interaction probe-sample in air simplest conditions of operation. 

In previous study lytic cycle phages directly determined by AFM method which detected morphology and 3D topography changes of bacterial cells and decrease of cell height. In our study we investigated not only interaction MRSA-phages by turbidity assay but also ultra-structural changes of phage-treated MRSA strains currently detected by AFM method in terms of MRSA strains morphology, roughness parameter, 3D topography, cell height, and FFT parameter (17-21).

Our aim is detect the lytic activity bacteriophages through characterization of ultra-structural of MRSA cell wall in phage-treated MRSA using AFM technique. We directly detect lytic activity bacteriophages against MRSA strains with changes of morphology, roughness parameter, 3D topography and cell height.

## Materials and Methods

***Bacterial cells preparation***

MRSA ATCC as positive control, and MRSA bacteria previously were isolated from the blood of a six-month-old new-born at tertiary Bouali Sina Hospital in Mazandaran province, Iran, were examined by conventional microbial tests such as: Gram stain, tube coagulase, catalase, DNase, and mannitol fermentation test.

***Preparation of bacteriophages***

Sewage sample was collected from septic tank at the tertiary pediatric Bouali Sina Hospital in Mazandaran province, Iran. The sample was transported to the laboratory and stored at 4 ^°^C. The sample was centrifuged 11000 g, 5 min, then was filtered through a 0.22 µm filter. An equal volume of filtered sample was added to the MRSA ATCC No.33591 strain OD_600 nm_= 1 (22, 23). This combination was incubated in shaker incubator 150 rpm, overnight, at 37 ^°^C. 

**Figure 1:**
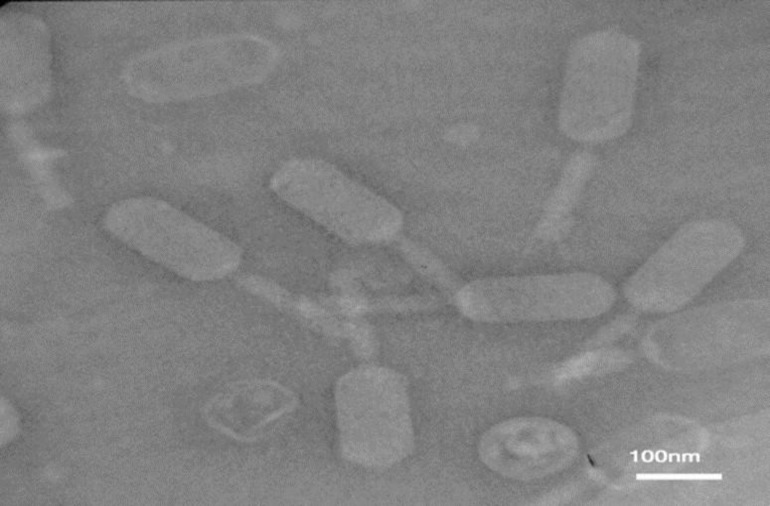
Electron micrographs of the family *Siphoviridae* phage serogroup A. Negatively stained with 2% uranyl acetate (pH=4-4.5), voltage 150 Kv, the scale bar represents 60 nm

**Figure 2:**
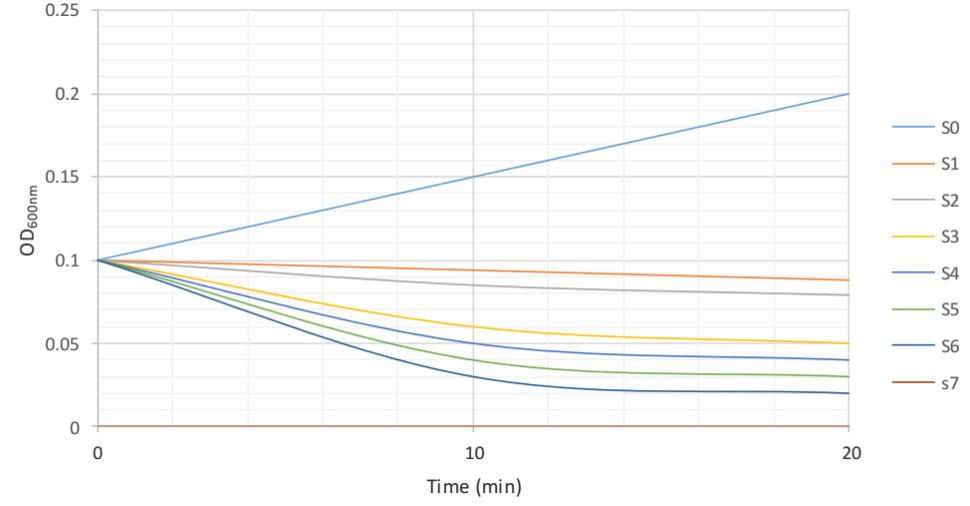
Turbidity assay of methicillin resistant *Staphylococcus aureus* (MRSA) ATCC 33591 in the presence of lytic phage

**Figure 3:**
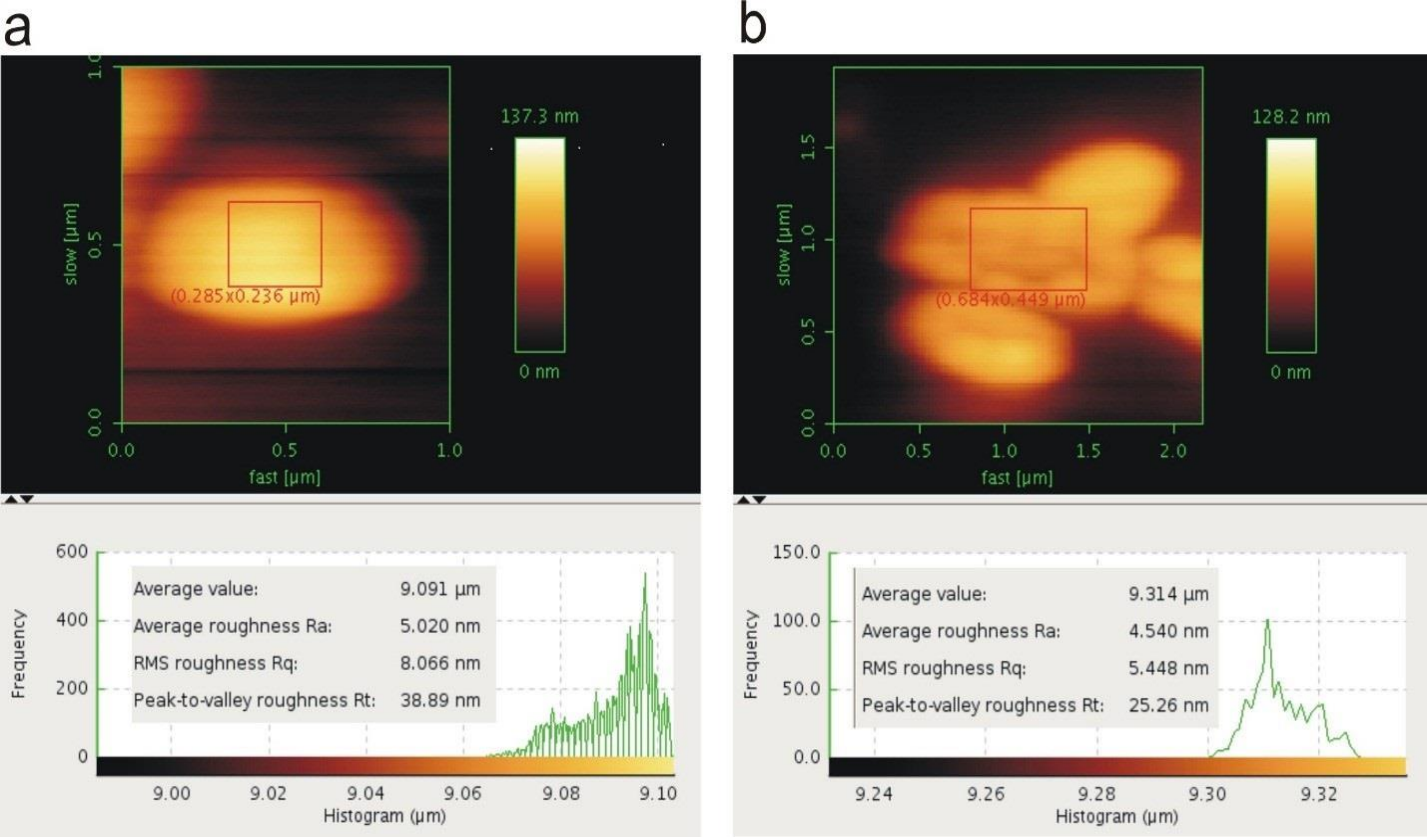
(a) Atomic force microscopy (AFM) micrograph of Methicillin Resistant *Staphylococcus aureus* (MRSA) cells (1.5×10^3^ CFU/ml) with the inset of roughness parameters at the bottom; (b) AFM micrograph of MRSA cells (1.5×10^3^ CFU/ml) affected by lytic bacriophages (10×10^8^ PFU/ml) with the inset of roughness parameters at the bottom. The micrographs have been obtained and analyzed using JPK atomic force microscope with 150 Hz IGain, 0.0048 PGain, and 1.0 V set point via a JPK NanoWizard control

**Figure 4:**
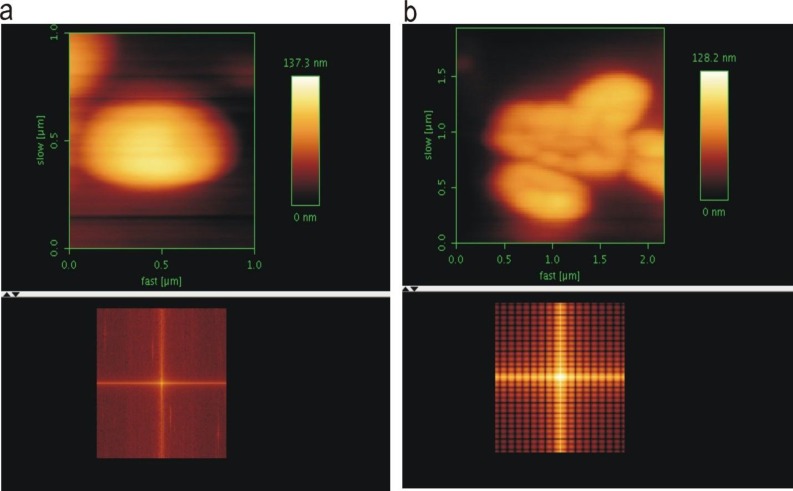
Atomic force microscopy (AFM) micrographs and fast Fourier transform (FFT) histograms of methicillin resistant *Staphylococcus aureus* (MRSA) cells (1.5×10^3^ CFU/ml) before (a) and after (b) interaction with lytic bacriophages (10×10^8^ PFU/ml)

**Figure 5:**
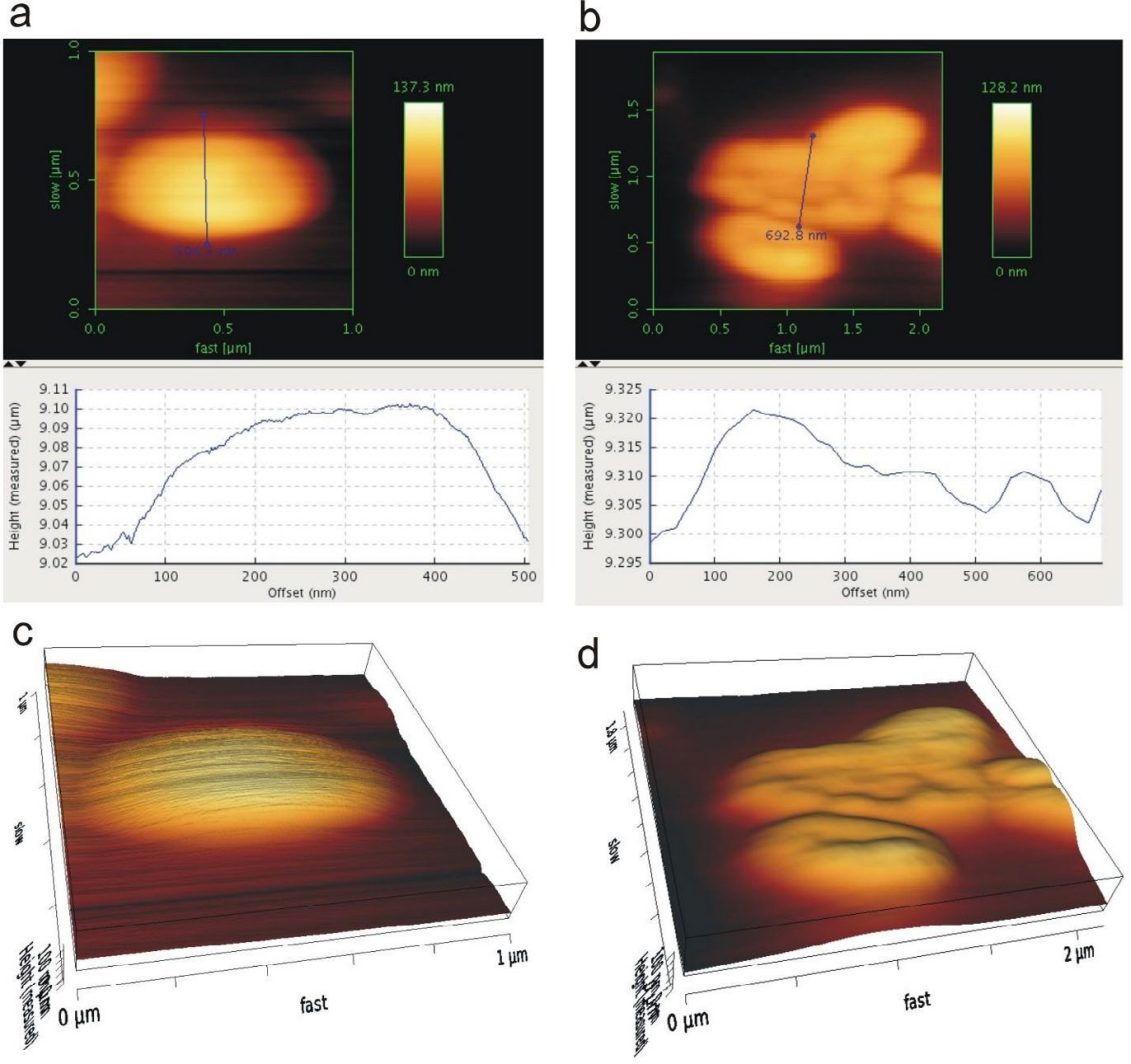
Two and three dimensional atomic force microscopy (AFM) micrographs of methicillin resistant *Staphylococcus aureus* (MRSA) cells (1.5×10^3^ CFU/ml) before (a) and after (b) interaction with lytic bacriophages (10×10^8^ PFU/ml) supported by size scaled topographic graphs

**Table.1:** Effective dose lytic phage against Methicillin Resistant *Staphylococcus aurous* (MRSA) bacteria

Sample Name	**S0**	**S1**	**S2**	**S3**	**S4**	**S5**	**S6**	**S7**
MRSA Count (CFU/ml)	1.5×10^8^	1.5×10^8^	1.5×10^7^	1.5×10^6^	1.5×10^5^	1.5×10^4^	1.5×10^3^	0
Lytic phages (PFU/ml)	0	10×10^8^	10×10^8^	10×10^8^	10×10^8^	10×10^8^	10×10^8^	0

***Double-layer plaque assay***

To detect MRSA bacteriophages titer DLA assay was performed. Ten-fold dilutions of isolated phages (10×10^8 ^PFU/ml) were prepared then of each dilution except Tubes No 9 and 10 were negative and positive controls, 100 µl was removed and added to 100 µl MRSA ATCC 33591 suspension (OD _600 nm_= 0.8). This solutions were added to 3 ml of top agar and mixed gently, then were poured into plates contains of bottom agar. The plates were incubated at 37 ^°^C, overnight. The next day the number of plaques were counted. After overnight the plaques formed were counted and phages titer were calculated (22, 23).

***Determination of morphology isolated phages with TEM***

Isolated phages were characterized by TEM. Bacteriophages were concentrated by centrifugation at 25000 g for 60 min, followed by two washes in 0.1 M neutral ammonium acetate. 

Purified bacteriophages were deposited on carbon-coated copper grids and stained with 2 % uranyl acetate (pH= 4-4.5). After staining, phages were observed on a Philips CM 300 electron microscope at 150 Kv (22, 23). 

***Sample preparation for AFM***

The bacteria samples divided in negative control, positive control, and experimental control groups. The negative control bacteria were incubated 24 hr at 37 ^°^C without phages and 10 μl of samples (OD_600 nm_= 0.8) were removed, fixed on the slide, then dried at room temperature (25 ^°^C) (21-23).

In order to investigate lytic activity of phages, turbidity concentration method was used. The MRSA ATCC 33591 in Luria-Bertani (LB) medium was inoculated and was shacked in shaker incubator at 37 ^°^C until the OD_600 nm_ reached 0.1 (1.5×10^8^ CFU/ml). Serial dilutions of the MRSA bacteria were prepared in 6 tubes, the tubes numbered 0 and 7 were positive control and negative control respectively. The ratio of 1:1 diluted MRSA ATCC 33591 with phages (10 ×10^8^ PFU/ml) were combined. At 10 min intervals the concentration of MRSA ATCC 33591was studied in presence of phages at OD_600 nm_. Similar to the above method was performed with MRSA which were isolated from the blood of a six-month-old new-born at tertiary Bouali Sina Hospital (21-23). 

***Atomic force microscopy***

The slides were studies using contact mode with JPK-AFM, with 150 HZ 1Gain, 0.0048 PGain and 1.0V set point via a JPK Nano Wizard Control. The cantilever was ACTA-10 probe model (material: silicon, N-type, 0.01-0.025 Ω/cm). Rough data were graphically processed with the JPK Nanoanalyzer Software (24).

## Results

***Hospital strain confirmation***

The microbial tests confirmed MRSA isolation from the blood of a six-month-old new-born with septicaemia.

***Characterization of lytic activity bacteriophages***

Formation plaques indirectly showed lytic activity of MRSA bacteriophages. The results showed formed plaques with 9-14 μm diameter and phages titer calculated as 10×10^8^ PFU/ml. 

***TEM characteristics of bacteriophages***

The results of electron microscopy with negatively stained, 2% uranyl acetate (pH= 4-4.5), and voltage 50 Kv showed one group phages isolated. Phages with icosahedral head and no contractile tail lengths (300 nm) belonged to the family *Siphoviridae* (order *Caudovirales*) serogroup A (Figure 1).

***Indirect investigation of lytic activity MRSA bacteriophages by turbidity concentration method***

In order to investigate the lytic activity of phages against MRSA ATCC 33591 and MRSA isolated from patient specimen, the turbidity concentration method was used. The results showed after 10 min, concentration of MRSA, ATCC 33591 in samples S3 (1.5×10^6^ CFU/ml), S4 (1.5×10^5^ CFU/ml), S5 (1.5×10^4^ CFU/ml), S6 (1.5×10^3^ CFU/ml) decreased 2-log, 3-log, 4-log, and 5-log respectively. Samples S2 (1.5×10^7 ^CFU/ml), and S1 (1.5×10^8^ CFU/ml) decreased 1-log. After 20 min, the concentration of MRSA ATCC 33591 in samples S1, S2, S3, S4, S5, and S6 were not significantly changed (Figure 2) (Table 1).

***Direct investigation lytic activity MRSA bacteriophages using AFM***

The results of AFM directly showed lytic activity bacteriophages after 10 min incubation of phages with MRSA ATCC 33591 and isolated MRSA strain from patient sample. Infected MRSA strains determined by significant changes in bacterial morphology such as destruction of the cell wall, decrease of cell height, and loss of their shape. AFM micrographs analyzed roughness parameters at the MRSA strains cell wall (Figure 3A) and the results showed the average roughness (Ra) and RMS roughness (Rq) of the phage-treated MRSA and MRSA ATCC 33591 significantly more than results of the untreated MRSA. In addition, peak-to-valley roughness parameter was decreased for the phage-treated MRSA cells when compared to that for the untreated MRSA (Figure 3B). The most changes of bacterial morphology, destruction of cell wall, decrease of cell height, and loss of their shape at phage-treated MRSA ATCC 33591 were S1 (1.5×10^8^CFU/ml), S2 (1.5×10^7^CFU/ml), S3 (1.5×10^6^ CFU/ml), S4 (1.5×10^5^ CFU/ml), S5 (1.5×10^4^ CFU/ml), S6 (1.5×10^3^ CFU/ml) respectively .

***AFM fast Fourier transform (FFT) characteristics of phage-treated MRSA***

AFM micrographs of MRSA strains ultra - structurally analyzed using FFT (Figure 4A)**. **The FFT histogram of phage-treated MRSA strains markedly illustrated changings on their surfaces of cell-wall due to the lytic activity of bacteriophages. The holes at the cell walls were depicted as the multiple crosses at the AFM FFT histogram of the MRSA bio molecularly affected by the phages (Figure 4B).

Three-dimensional (3D) AFM micrographs of phage-treated MRSA cells indicated topographic changings at their cell-wall surfaces when compared with the 3D micrograph from the untreated MRSA cells (Figure 5A) (Figure 5B). Also the topographic informations shown changing the height and size of phage-treated MRSA cells. The cell height of phage-treated MRSA strains were decreased and loss their shape.

## Discussion

In this study *Siphoviridae* bacteriophages were isolated from sewage at the tertiary hospital. Phages titer was calculated 10 ×10^8^ PFU/ml by DLA assay. Interaction of MRSA -phages by AFM technique directly investigated. Currently lytic activity phages was detected by turbidity assay. The results showed after 10 min phages (10×10^8 ^PFU/ml) decreased 5-log of MRSA ATCC 33591 S6 (1.5×10^3^ CFU/ml) which in these conditions AFM micrographs showed the most changes of MRSA ATCC 33591 S6 (1.5×10^3^ CFU/ml) morphology, roughness parameter, 3D topography, decrease cell height, and FFT.

 AFM is the most efficient tool for studying interaction bacterium-phage among the other high resolution types of microscopy. The advantages of AFM include: easy sample preparation, operation in ambient or aqueous conditions, obtain 3D topographical images, evaluate the mechanical properties of sample, and samples of non pretreatment with metal coating but in EMs methods samples pretreatment with metal coating is needed in which leads to change of sample properties.

In study by Abdulamir *et al.*, isolated phages from sewage at hospital and cattle waste and lytic activity of isolated phages on MRSA biofilm investigated by SEM. SEM screening used to only visualize morphology of biofilms in presence of phages. but in our study AFM micrographs demonstrated not only phage-treated bacteria morphology but also roughness, 3D topographical data, Fourier transform (25, 26). 

AFM technique monitor interaction phage-bacterium in different infected stages from adsorption on bacterial cell surface to release of new phages. AFM directly detect the lytic activity of phages with decrease of bacterial height, loss of bacteria shape with numerous newly formed phages as clouds near the host cell and destruction of host cell (18-20). We directly scanned lytic activity of phage against MRSA in the air condition as the simplest conditions of operation with high resolution imaging.

 In this study AFM micrographs shown MRSA morphology changes after 10 min. The comparison shape and size infected MRSA with non-infected MRSA directly approved the lytic activity of isolated phages. The results showed after 10 min the most changes of morphology, roughness parameter, 3D topography, decrease cell height, and FFT in phage-treated MRSA S6 (1.5×10^3^ CFU/ml) which lytic activity phages (10×10^8 ^PFU/ml) decreased 5-log of MRSA S6 (1.5×10^3^ CFU/ml) .

In study Dubrovin *et al.*, reported lytic activity of bacteriophages A157 against Gram-negative *Escherichia coli* and *Salmonella enteritidis* and Gram-positive *Bacillus thuringiensis* by AFM. The results shown the effect of bacteriophages on the Gram-negative *E. coli* and *S. enteritidis*. Also similar to our study, significant changes in bacterial morphology such as destruction of the cell wall, decrease of cell height, and loss of their shape was observed (27, 28).

 Increasing roughness of infected MRSA is directly reflected from phage adsorption on the bacterial cell surface to destroy the cell wall during the infection process (29).

Also our results shown great potential of using other modes of AFM operation for directly study phage-bacterial infection process such as force spectroscopy and different types of affine imaging in solution. Sample preparation play an important role and can be crucial for obtaining adequate results. Insufficient sample purification can lead to hardly interpreted AFM images, or very small initial ratio of phages/bacteria concentration may lead to hardly find in AFM images.

In our study lytic activity of phages in phage-treated bacteria directly assayed by AFM but adsorption stages on bacterial surface and its effect on bacterial cell wall did not investigated.

## Conclusion

According to our knowledge, this study for the first time directly showed ultra-structural characterizations of MRSA bacteria cell-wall after interaction phage-bacterium by AFM. We directly investigated interaction of phage-bacterium and studied lytic activity bacteriophages. Analysis and comparison of shapes, sizes, and 3D topography, roughness, bacteria height in phage-treated MRSA bacteria with positive control group confirm lytic activity of bacteriophages. AFM micrographs directly present the results of turbidity assay.
